# The Nucleic Acid Database: new features and capabilities

**DOI:** 10.1093/nar/gkt980

**Published:** 2013-10-31

**Authors:** Buvaneswari Coimbatore Narayanan, John Westbrook, Saheli Ghosh, Anton I. Petrov, Blake Sweeney, Craig L. Zirbel, Neocles B. Leontis, Helen M. Berman

**Affiliations:** ^1^Department of Chemistry and Chemical Biology, Center for Integrative Proteomics Research, Rutgers, the State University of New Jersey, 174 Frelinghuysen Road, Piscataway, NJ 08854-8076, USA, ^2^Department of Chemistry and Center for Biomolecular Sciences, Bowling Green State University, Bowling Green, OH 43403, USA and ^3^Department of Mathematics and Statistics, Bowling Green State University, Bowling Green, OH 43403, USA

## Abstract

The Nucleic Acid Database (NDB) (http://ndbserver.rutgers.edu) is a web portal providing access to information about 3D nucleic acid structures and their complexes. In addition to primary data, the NDB contains derived geometric data, classifications of structures and motifs, standards for describing nucleic acid features, as well as tools and software for the analysis of nucleic acids. A variety of search capabilities are available, as are many different types of reports. This article describes the recent redesign of the NDB Web site with special emphasis on new RNA-derived data and annotations and their implementation and integration into the search capabilities.

## INTRODUCTION

The Nucleic Acid Database (NDB) was founded in 1991 to assemble and distribute structural information about nucleic acids (1). In addition to the primary structural data that are contained in the archival Protein Data Bank (PDB) (2), the NDB contains annotations specific to nucleic acid structure and function, as well as tools that enable users to search, download, analyze and learn more about nucleic acids. NDB is thus a value-added database providing services specifically for the nucleic acid community.

**Figure 1.**
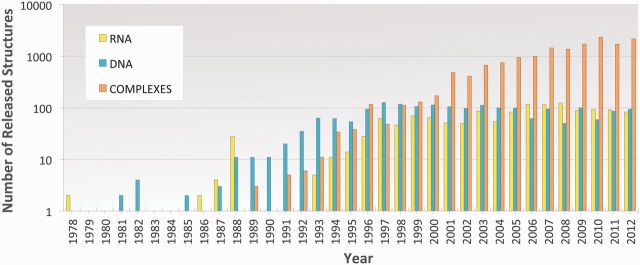
Growth of the number of nucleic acid structures in NDB. The total number of structures released in log scale per year for RNA (yellow), DNA (blue) and protein-nucleic acid complexes (orange) is shown.

When the NDB was first established, the focus was on DNA structural biology. As more RNA structures have been determined (Figure 1), tools and annotations were developed to address the features of these molecules.

The NDB seeks to be a central source for nucleic acid structural information and annotations that evolves with the science. In this article we describe the recent redesign of the NDB Web site with special emphasis on new RNA-derived data and annotations and their implementation and integration into the search capabilities.

## NDB ACCESS

**Figure 2.**
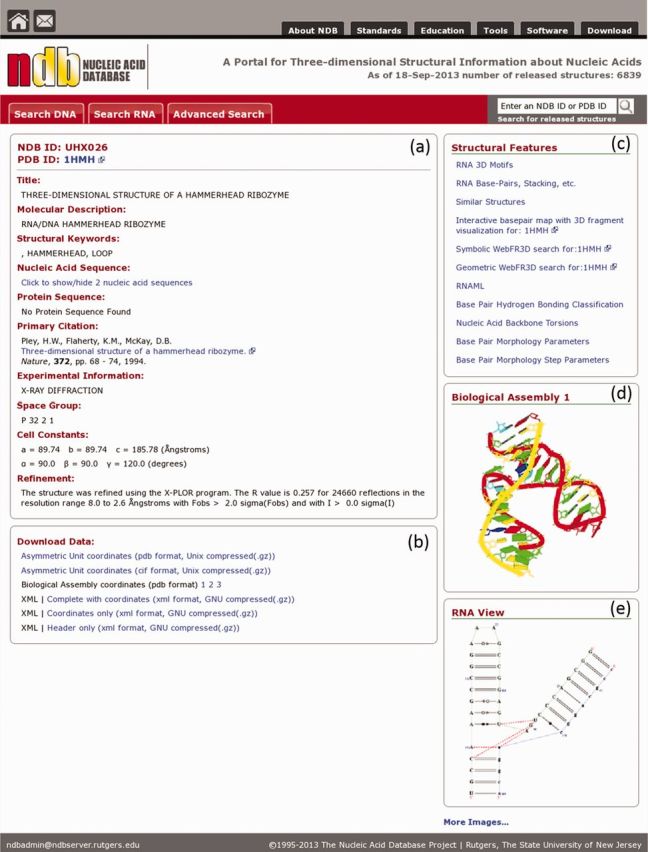
Structure summary report showing the gray and red persistent headers, and individual sections for (**a**) primary structural information, (**b**) atomic coordinate and experimental file download, (**c**) derived structural data, (**d**) images and (**e**) for RNA structures an additional RNA view image.

All available NDB resources can be accessed through two persistent headers available on top of all the pages in the Web site. The first persistent header seen in gray in Figure 2 consists of six tabs: About NDB, Standards, Education, Tools, Software and Download.

### About NDB

Information about the project including a site map.

### Standards

Information about the standard reference frame for the description of nucleic acid base pair geometry (3); ideal geometries for bases and sugars (4,5); DNA/RNA topology and parameter files for refinement of structures (6); mmCIF resources (7); PDBML resources (8); and a link to the RNA ontology consortium (9,10).

### Education

Introduction to nucleic acids; definitions of terms used in the Web site; nucleic acid–related features from PDB-101, an educational component of RCSB PDB (11); and links to other educational activities and sites.

### Tools

Recently added features include the RNA 3D Motif Atlas (12); nonredundant (NR) lists of RNA-containing 3D structures (13); the RNA Base Triple Atlas (14), a tool to perform nucleotide to nucleotide alignment of two RNA 3D structures (R3D align) (15,16); and a server for finding, aligning and analyzing recurrent RNA 3D motifs (WebFR3D) (17). These tools are also highlighted in the ‘Featured Tools’ section of the homepage. Other NDB tools include a secondary structure similarity search (QPROF) (18); an RNA 2D structure viewer (RNA View) (19); and an option for the analysis and visualization of nucleic acid structures (w3DNA) (20). Links to a number of other external resources for both RNA and DNA are provided.

### Software

Downloadable software includes a geometric and symbolic 3D motif search (FR3D) (21), visualization of secondary structure (RNA View) (19) and visualization of 3D structures (3DNA) (20). Links to software packages from other groups have also been provided, including software for statistical folding of nucleic acids and studies of regulatory RNAs (Sfold; http://sfold.wadsworth.org) (22,23), a visualization applet for RNA (VARNA) (24) and the UNAFold web server (25,26).

### Download

The coordinate and experimental files for all the structures in NDB are available under the ‘download’ tab. A mapping of PDB ID to NDB ID for released entries is also available.

The second persistent header shown in red in Figure 2 includes the search options (simple search, advanced search and ID search) that are described in a subsequent section.

## NDB DATA CONTENT

The NDB contains primary structural information about nucleic acid containing structures obtained from the PDB as well as classifications and derived data. Manually annotated nucleic acid classifications as well as derived and calculated data regarding structural features of RNA are managed separately from the primary structure entries; these data are recorded and stored as external reference files (ERFs).

### Primary structural information

The primary data obtained from the corresponding PDB structure entries include experimental files, identifiers, structural descriptions, citations, crystal data, coordinate information and details regarding crystallization, data collection and structure refinement (Table 1).

**Table 1.** NDB primary content acquired from PDB and its description

Primary content	Description
Coordinate information	Atomic coordinates for the asymmetric and biological unit
Experimental files	Structure factor files, NMR restraints
Identifiers	NDB ID, PDB ID
Structural description	Sequence, description of asymmetric and biological unit, base pairing, mismatches, modifications
Citation	Title, authors, journal, volume, year, pages, PUBMED id, DOI
Crystal data	Cell parameters and space group information
Crystallization details	Method, temperature, pH and crystallization condition
Data collection information	Radiation source, detector, wavelength, temperature, resolution, number of reflections, Rmerge
Refinement details	Method and programs used, resolution, R-factor, number of reflections, refinement of temperature factors and occupancies

### Nucleic acid classifications

Annotations specific to nucleic acids and the molecules to which they are bound are provided (Table 2). Nucleic acid annotations include nucleic acid type and conformation, structure description and secondary structure. Some functional information about the bound proteins as well as drug binding modes is also offered.

**Table 2.** Nucleic acid classifications stored in the ERFs and an explanation of its content

NDB classification	Description
Descriptor	Description of the contents of asymmetric unit
Conformation type	Structural conformation type (A/B/Z/RH/U)
Secondary structure information	Secondary structure classification (loop, double helix, triple helix and quadruple helix)
Nucleic acid type	Type of nucleic acid (ribozyme, riboswitch, etc.)
Protein type	Protein class (enzyme/structural/regulatory/other) and type
Drug binding mode	Name and binding type of drug

### Derived data

Derived structural features such as bond distances, angles, torsions and base morphology (20,27) are calculated from the coordinate data and stored in the searchable database (Table 3).

**Table 3.** List of the calculated derived data and its contents

Derived data	Content
RNA motifs	Internal and hairpin loop motifs in RNA structures
NR data	NR list of RNA containing structures
RNA 3D interactions	Base pair parameters, base phosphate interactions, base stacking interactions
Distances and angles	Covalent bond lengths and angles, valence bond lengths and angles
Torsions	Backbone and side-chain torsions
Base morphology	Base morphology and base pair step parameters

We have recently added derived information on RNA structural features including pairwise nucleotide interactions for each RNA structure, equivalence classes and NR sets of RNA structure files and RNA 3D motifs extracted from structures.

#### RNA pairwise interactions

Pairwise interactions between RNA nucleotides are annotated using FR3D (21) for RNA base pairing and base stacking interactions and as described in (28) for base phosphate interactions. These annotations form the basis for the RNA 3D Motif Atlas and the RNA Base Triple Atlas. In addition, we provide statistics on pairwise interaction frequencies. These data may prove useful to modelers and other computational scientists interested in determining characteristics of structured RNAs.

#### Equivalence classes and NR sets

RNA-containing entries are grouped into ‘equivalence classes’ of structures that share the same, or nearly the same, sequence and geometry, as described in (13). These equivalence classes are computed every week so that new additions to the 3D structure database are quickly reflected. Generally, different structures of the same RNA from the same organism appear in the same equivalence class, while structures of homologous RNAs from different organisms appear in distinct classes. For example, NR_4.0_00834.10 is the accession number for the equivalence class of *Escherichia coli* large subunit (LSU) ribosome structures, as of 20 July 2013. This equivalence class has 74 members. When gathering statistics across many RNA structures, it is not appropriate to include all 74 *E. coli* LSU structures as if they provide independent data points. Therefore, one structure with the largest number of FR3D-annotated base pairs per nucleotide is chosen to represent this equivalence class. A NR set of RNA 3D structures results from using the representative structure from each equivalence class. The NDB home page provides links to lists of equivalence classes and the structures contained in the current NR set. NDB search functions allow the user to limit results to include only one structure from each equivalence class.

#### RNA 3D Motif Atlas

The RNA 3D Motif Atlas, linked to from the NDB home page, is an organized collection of internal and hairpin loops extracted from the NR set of 3D structures (12). Individual motif instances are organized into motif groups, containing all instances that share the same pattern of base pairing interactions and overall geometry. The Atlas is updated automatically every 4 weeks.

The manually annotated nucleic acid classifications as well as derived and calculated data regarding structural features of RNA are managed separately from the primary structure entries; these data are recorded and stored as ERFs.

## SEARCH CAPABILITIES

**Figure 3.**
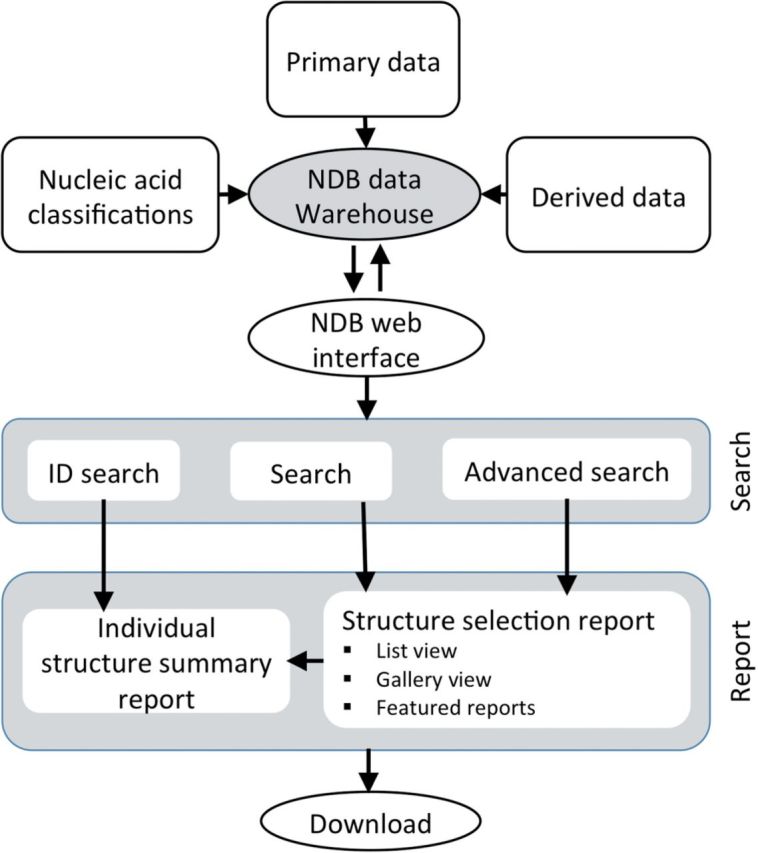
Schematic representation of the data flow to and from the data warehouse and the web interface.

The NDB data flow is depicted in Figure 3. To facilitate search and reporting functions, the NDB stores primary structural data, classification data and derived data in a relational database. The database content includes the primary structural information acquired from the PDB, and the ERFs containing nucleic acid classifications, calculated derived data and additional derived data on RNA structural features. Search, reporting and download functionalities are provided by a web interface. Search results are returned either as individual structures or groups of structures depending on the query. Search results are linked to a variety of reporting features including predefined feature reports, navigation to individual structure summary reports that then permit the download of primary data files, derived data and molecular images.

Three search options are available from the secondary persistent header (red in Figure 2): ID search, search and advanced search. These search options can also be accessed in the ‘Search Structures’ section of the homepage.

### ID Search

The ID search accepts either an NDB ID or PDB ID as input and the result is the individual Structure Summary for the entered structure.

### Search

Search is available separately for DNA and RNA structures. Users can create a set of entries (results) by constraining particular predefined data attributes in several categories. Following a preliminary search, users can add selection constraints from additional categories to further narrow their searches.

#### DNA search

Selection options are organized under the headings polymer, protein function, structural features and experimental method:

- Polymer: Select structures based on polymer composition such as DNA only, protein–DNA complexes, drug–DNA complexes, hybrids, chimera and peptide nucleic acid mimetics.
- Protein function: Narrow the search based on the type of protein found in the protein–DNA complex: enzyme, structural protein, regulatory protein or other classification.
- Structural features: Find structures based on secondary structure and conformation types. Options include single-stranded, A-form, B-form or Z-form DNA double helices, triple or quadruple helices and other double helical structures.
- Experimental method: Select the experimental method used to determine the structure [X-ray crystallography, nuclear magnetic resonance (NMR)].

#### RNA search

The polymer, protein function and experimental method selection categories present options similar to those provided for DNA searches. Additional options are provided for RNA to restrict search results to the representative structures belonging to the NR data set. The RNA-specific selection categories include the following:

- RNA type: Search various RNA functional types, such as tRNAs, rRNAs, riboswitches or ribozymes.
- NR list: Restrict the RNA search to the representative members of equivalence classes that constitute the NR RNA structure set. This filtering dramatically reduces the number of structures returned by the search without diminishing the range of molecules represented and provides lists of structure that are more suitable for statistical analyses. Putting only the nonredundancy constraint on the query will result in a ‘nonredundant list’ of the best modeled structures (in terms of the number of base pairs per nucleotide) at the specified resolution threshold.

### Advanced search

The Advanced Search allows users to compose queries combining multiple selection constraints using logical operators. Selection constraints are organized into the following categories: structure content, experimental information, experimental details, citation, RNA 3D interactions, RNA 3D motifs, sequence, nucleic acid modifications, protein binding type and nucleic acid conformation type.

- Structure content: Restrict searches based on the presence or absence of a type of molecule (DNA, RNA, protein, hybrid molecules and drugs).
- Experimental information: Restrict searches by experimental method (X-ray/NMR) and by the availability/nonavailability of experimental files.
- Experimental details: Restrict searches by user-provided values for crystal cell dimensions and space group.
- Citation: Restrict searches to specific authors, publication years or PDB/NDB ID.
- RNA 3D interactions: Define searches according to the presence and relative frequencies of any of the base pair, base phosphate and base stacking interactions. The relative frequencies of interactions are calculated with respect to the total number of interactions of that type occurring in the structure.
- RNA 3D motifs: Restrict searches to structures that contain certain named RNA motifs.
- Sequence: Restrict searches to a specific nucleotide sequence pattern present in the structure and a range of overall sequence length.
- Nucleic acid modifications: Constrain searches based on the presence or absence of chemical modifications in bases, sugars or phosphate.
- Binding type: Restrict searches of protein complexes according to type of protein, protein function and type of nucleic acid to which it binds.
- Nucleic acid structural conformation type: Narrow searches according to presence of structural features such as bulges, three-way junction, non–Watson-Crick base pairing along with strand description and conformation type.

For each of the search criteria, the options to explicitly select, deselect and ignore are available as Y, N and ignore, respectively, with the default being ignore. When combining two or more search queries, logical operators AND (to restrict results) or OR (to combine results) are available with the default being AND. For example, to get all NMR structures that have base modifications, select NMR AND base modification by clicking ‘yes’ next to each of them and choosing the logical operator AND. This search returns only those structures that satisfy both criteria. The results of each search appear in a new window and include NDB ID, PDB ID, title, authors, initial deposition and release dates, and links for further information.

## REPORTING CAPABILITIES

The results of a structure selection search are presented as a structure selection report, an image gallery, or as one of a set of predefined feature reports. A detailed structure summary report is available for every structure.

### Structure selection report

The structures selected by a search are displayed in a tabular report containing the essential features for the structure selection, including the title, authors, citation and release date of the structure, the type of experiment, type of structure, a link to the equivalence class to which it belongs and the representative structure of that class, as well as a structure image. The structure selection report is also available as a gallery of structure images with their accession codes. In both the gallery and summary reports, the structure accession code provides a link to a more detailed structure summary report for each selected structure.

### Structure summary report

Each structure in NDB has its own individual summary page containing information relevant to that structure. Data are presented in the structure summary page in four sections: (a) primary structure information, (b) downloads, (c) derived structural data and (d) images (Figure 2).

The main (primary structure information) section (Figure 2 Section a) holds the entry title, sequence, citation, experimental details, refinement information and various structural descriptions. The atomic coordinates (asymmetric and biological unit files), structure factors and NMR restraint information are available in the ‘Download Data’ section. The ‘Structural Features’ panel in the upper right of the window (Figure 2 Section c) presents links to derived information including hydrogen bonding, torsions and base morphology, and step parameters. Below the Structural Features panel, the contents of the asymmetric unit or the biological assembly model is shown as a 3D image (Figure 2 Section d). For RNA entries, an RNA View image showing 2D base pairing is also provided (Figure 2 Section e). Additional images of biological assemblies, crystal packing and ensemble images are available under the ‘more images’ link.

**Figure 4.**
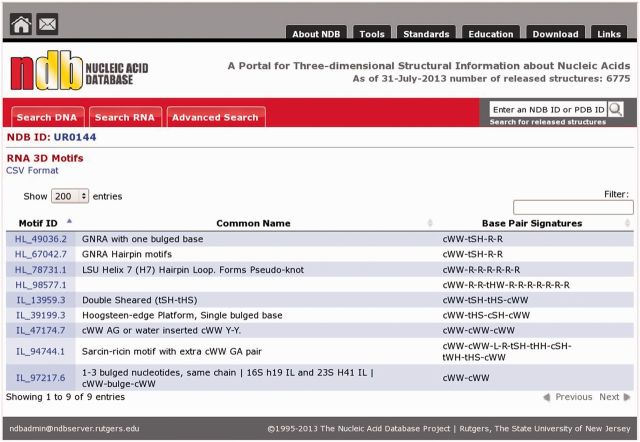
The structural features section of the summary page contains a summary of motif information for RNA structures. The ‘RNA 3D Motif’ summary provides a list of internal and hairpin loop motifs found in the structure along with their base pair signatures and common names.

For RNA-containing structures, many additional ‘Structural Features’ are now available. For those NDB structures that are the representative of their equivalence, the RNA 3D Motifs page lists all internal and hairpin loops in the structure, and links to the corresponding entries in the RNA 3D Motif Atlas (12). The base pair signature of the motif is provided, and, when available, the common name of the motif (Figure 4).

The ‘Structural Features’ section also links to annotations of RNA base pairs, base stacking, and base phosphate interactions in a tabular form, as annotated by the FR3D program suite (21) (28). Because some structures are large, the list of interactions can be filtered to view only interactions of a given type, for example, *cis*-Watson-Crick/Hoogsteen (cWH) or trans Hoogsteen/Sugar Edge (tHS) base pairs. At the bottom of the pairwise interaction page is a summary of the counts and relative frequencies of the different types of interactions. In the pairwise interaction list, each RNA nucleotide is identified by a unique unit ID; this is a text string constructed from the PDB ID, the model number, the chain, the RNA base or amino acid and the residue number. For example, the annotation 1S72|1|9|U|99 1S72|1|9|G|83 cWW unambiguously refers to the GU cWW base pair made between two nucleotides in chain 9 of model 1 of PDB file 1S72. Unit IDs provide a way to uniquely and unambiguously refer to any unit in any structure, a need identified by the RNA Ontology Consortium (9,10). By clicking the ‘Similar Structures’ link in the ‘Structural Features’ panel, one can reach new pages listing structures belonging to the same equivalence class.

The ‘Structural Features’ section also provides a link out to interactive visualizations of the base pairs in RNA structures, in the form of RNA circle diagrams (29). The interactions are displayed as clickable arcs colored by base pair type, with all nucleotides in the structure arranged around a circle. Moreover, for certain structures, a more conventional secondary structure diagram is available. In either case, the user can select base pairs to display by type, mouse over the interaction arcs to see the participating nucleotides, and select pairs or regions to visualize in 3D in an adjoining Jmol window. Finally, links are provided in the ‘Structural Features’ section to facilitate WebFR3D searches within the current structure.

### Featured reports

Featured reports are available for the result set of any advanced search query. A predefined set of reports is provided: NDB status, cell dimensions, citation, refinement data, backbone torsion, base pair and base step parameters, descriptor, sequence and RNA motifs. The NDB status report containing NDB and PDB ID’s, structure title, authors, deposition and release dates is the default report for any advanced search query. The content of each of these reports is described in Table 4. All these reports can be exported as spreadsheets for further analysis.

**Table 4.** The list of ‘featured reports’ available for advanced search queries and the content of each of the report

Report	Content
Motifs	Motif ID with common name and base pair signature
NDB status	Default report with structure ID’s, title, authors, deposition and release dates
Cell dimensions	Crystallographic cell parameters and space group
Citation	Author, title, journal, volume, page numbers, year
Sequences	Nucleic acid sequence and molecular description
Descriptor	Structure ID’s with entry title
Refinement data	R-factor, resolution, number of reflections, program used for refinement
NA backbone torsions	Sugar-phosphate backbone torsion angles
Base pair parameters	Global base pair parameters calculated using standard reference frame (3)
Base pair step parameters	Local base pair step parameters calculated using standard reference frame (3)

## DATA RETRIEVAL

The atomic coordinates for the asymmetric unit and for all biological assemblies are available in the ‘downloads’ section of the corresponding NDB structure summary report and are provided in PDB as well as mmCIF formats. The asymmetric unit coordinates are also available in PDBML (XML) format for the complete file or for the header and the coordinate sections separately. Structure factor data are provided in mmCIF format, and NMR restraint information are available in deposited program format. All these files are updated weekly during database update and are downloadable from the NDB ftp server at ftp://ndbserver.rutgers.edu/NDB/.

## INFRASTRUCTURE

### Web service framework

In the new NDB site, we have transitioned from a reliance on pregenerated HTML pages to dynamically generated page content. Each page has been partitioned to load content sections on demand using AJAX protocol web services. A framework supporting REST style web service queries has been created to support dynamic content AJAX functionality. For instance, within the structure summary pages, RNA Motif and interaction classification statistics are obtained from asynchronous database queries, as these are requested by users viewing the summary page. Search summary and structure browsing pages are similarly generated using this dynamic protocol. The new framework has been implemented using Python language middleware and Apache web server FastCGI protocol request handling. Web pages rendered in HTML take full advantage of CSS and JavaScript.

### Database server infrastructure

In the new release of the NDB Web site, the proprietary relational database engine IBM DB2 has been replaced with the open source MySQL database engine. A new Python language middleware has been developed to support NDB loading operations, query construction and report generation using the MySQL storage engine. Moving to the MySQL server has dramatically improved the portability of the NDB site and simplified database maintenance and administration. The new database infrastructure facilitates site replication and synchronization, allowing us to support production, beta and development instances. This capability has enabled rapid implementation and testing of new project functionality.

### Pipeline for RNA structure annotations

The pipeline to exchange primary structure data between PDB and NDB has been in place for many years. A new pipeline has been developed for the regular exchange of additional RNA 3D structural annotations created by the RNA group at Bowling Green State University (BGSU); moreover, this pipeline can be used for data exchange from other research groups as needed in the future. The annotations from BGSU include the assignment of NR representative RNA structure and associated equivalence classes, RNA 3D Motif assignments and RNA base pairing, base phosphate and base stacking interactions. These new RNA annotations are added to the NDB database as part of each weekly update.

### Portability, maintenance and administration

The source code supporting the redesigned NDB web resource has been organized into a file system that allows all of the source components of the site to be managed by the revision control system, Subversion (http://subversion.tigris.org/). The use of Subversion has simplified the management of the code base, enabling rapid deployment, synchronization and simultaneous development by multiple programmers. This in turn has dramatically improved the portability and simplified the administration of NDB applications on multiple servers.

## CONCLUSIONS

The recent redesign of the NDB highlights the improvements made in data content including annotations and derived data and their presentation. The entire Web site has been revamped to improve the query and reporting capabilities. Annotations of RNA-containing structures have been expanded significantly. The site is structured to facilitate the addition of more search options, annotations, visualizations and reports about nucleic acid containing structures in the future.

## ELECTRONIC ADDRESSES

NDB website: http://ndbserver.rutgers.edu
NDB Helpdesk: ndbadmin@ndbserver.rutgers.edu
FTP site: ftp://ndbserver.rutgers.edu/NDB/
Coordinate deposition to PDB: http://www.wwpdb.org/
RNA 3D Hub: http://rna.bgsu.edu/rna3dhub/
RCSB PDB-101: http://www.rcsb.org/pdb-101

## FUNDING

National Institute of General Medical Sciences (National Institutes of Health) [GM085328]; National Science Foundation [DBI 0829586]. Funding for open access charge: National Institutes of Health.

*Conflict of interest statement*. None declared.
